# Tumor-infiltrating lymphocytes in NSCLC: from immune surveillance to immunotherapy

**DOI:** 10.3389/fimmu.2025.1610998

**Published:** 2025-07-25

**Authors:** Haiyi Xue, Yilan Fan, Yi Li, Qian Zhao, Xuelu Zhang, Pei Zhao, Zhenjun Liu

**Affiliations:** ^1^ Department of Intensive Care Unit, Sichuan Clinical Research Center for Cancer, Sichuan Cancer Hospital & Institute, Sichuan Cancer Center, University of Electronic Science and Technology of China, Chengdu, China; ^2^ Department of General Medicine, MianYang Cancer Hospital, Mianyang, China

**Keywords:** lung cancer, tumor microenvironment, immune cells, immunotherapy, CD8 + T cell, B cell

## Abstract

Lung cancer, predominantly non-small cell lung cancer (NSCLC), remains a principal driver of cancer-related morbidity and mortality worldwide. Despite advancements in surgery, radiotherapy, chemotherapy, and targeted treatments, outcomes remain poor in advanced NSCLC. The tumor microenvironment (TME) exerts a critical influence on therapy responses. Within the TME, immune cells such as T and B lymphocytes, dendritic cells, myeloid-derived suppressor cells, tumor-associated macrophages, neutrophils, and natural killer cells can drive both pro- and anti-tumor processes. This review integrates their classification, phenotypic plasticity, and roles in NSCLC, highlighting key preclinical and clinical evidence while discussing pathogenesis, prognostic significance, and therapeutic potential. We also summarize the current immunotherapeutic strategies for advanced NSCLC, including first- or second-line regimens with immune checkpoint inhibitors alone or combined with chemotherapy, anti-angiogenic agents, or additional checkpoint inhibitors, and future directions. By elucidating the interplay between the NSCLC immune microenvironment and emerging immunotherapies, this review emphasizes the need for novel combination regimens and robust predictive biomarkers to improve clinical outcomes and extend survival in advanced NSCLC.

## 1 Introduction

Lung cancer remains the leading cause of cancer-related deaths worldwide, with non-small cell lung cancer (NSCLC) accounting for 80-85% of all cases (1, 2). Despite advances in surgery, radiotherapy, chemotherapy, and targeted therapies, the prognosis for advanced-stage NSCLC remains poor, with a five-year survival rate below 5% (3). Despite advancements in surgical resection, radiotherapy, chemotherapy, and targeted therapies, a majority of patients are diagnosed at locally advanced or metastatic stages, resulting in a poor prognosis (4). Immunotherapy has emerged as a novel treatment strategy for solid tumors, including lung cancer; however, most patients derive significant benefit from these interventions (4, 5).

The tumor microenvironment (TME) is composed of tumor cells, immune cells, cancer-associated fibroblasts (CAFs), signaling molecules, and the extracellular matrix (ECM) (6–9). It can be broadly categorized into the immune microenvironment, dominated by immune cells, and the non-immune microenvironment, primarily comprising CAFs (10). In the NSCLC TME, tumor cells exert profound effects on infiltrating immune cells, shaping an immunosuppressive milieu that promotes tumor progression (11). This review provides a comprehensive overview of the classification and recent advances in the study of immune cells within the NSCLC immune microenvironment.

## 2 Immune cells in the NSCLC tumor immune microenvironment

### 2.1 Antigen-presenting cells

#### 2.1.1 Dendritic cells

DCs, originating from the myeloid lineage, serve as central regulators of antitumor immunity and are the most potent local antigen-presenting cells (APCs) (12). The immune checkpoint molecule B7-H3 has been identified as an independent predictor of poor prognosis in NSCLC patients, with significantly upregulated expression observed in tumor-resident DCs of NSCLC patients (13). Cytokine-induced killer (CIK) cells in combination with dendritic cells (DC-CIK) have been shown to induce apoptosis in Lewis lung carcinoma cells, potentially through the downregulation of 14-3-3ζ and p-Bad proteins, thereby supporting the potential adjuvant role of DC-CIK in NSCLC combinatorial therapy (14, 15). Moreover, intratumoral injection of autologous dendritic cells engineered to express the CCL21 gene via an adenoviral vector elicited systemic antigen-specific immune responses, leading to increased CD8^+^ T cell infiltration and upregulation of intratumoral PD-L1 expression (16). In addition, non-small cell lung cancer cells downregulate the expression of costimulatory molecules such as CD80 and CD86 and pro-inflammatory cytokines including IL-12 and IL-23, while promoting the secretion of the anti-inflammatory cytokine IL-10 by CD1c^+^ dendritic cells, thereby impairing endogenous antitumor immunity (17). These findings provide a theoretical foundation for combining immunotherapy with *in situ* vaccination strategies, although further studies are needed to evaluate the efficacy of PD-1/PD-L1 checkpoint blockade in conjunction with DC-CCL21-based therapy.

#### 2.1.2 B cells

In NSCLC, tumor-infiltrating B cells (TIBs) are more abundant in tumor tissues than in adjacent non-tumor tissues, particularly in NSCLC (18). TIBs and CD4^+^ TILs are primarily located within tertiary lymphoid structures (TLSs), which correlate with improved prognosis in early-stage NSCLC, indicating TIBs’ antitumor role (19). Germain et al. (20) revealed that somatic hypermutation and class-switch recombination occur in TLS germinal centers in NSCLC, underscoring the prognostic significance of B cell density. Low CD20^+^ B cell and DCLAMP^+^ dendritic cell densities predict high mortality risk, suggesting their utility in therapeutic stratification. TIBs can present tumor antigens to CD4^+^ TILs, modulating their phenotypes into activated, antigen-related, and non-responsive subtypes, with activated TIBs linked to effector T cell responses (21). Beyond anti-CD20 therapies, B cell-targeted strategies remain preclinical, necessitating future research to enhance antitumor immunity. Conversely, TIBs also promote tumor progression by suppressing antitumor immunity, influenced by the TME. Regulatory B cells (Bregs) secrete IL-35 and TGF-β, inhibiting immune responses (22, 23). In autoimmune and transplantation contexts, B cell depletion impairs T cell function, while Bregs promote Treg phenotypes (24, 25). These findings highlight the dual role of TIBs in NSCLC and suggest TIB-CD4^+^ TIL interactions as potential immunotherapeutic targets.

### 2.2 Effector immune cells

#### 2.2.1 CD8^+^ and CD4^+^ T cells

Tumor-infiltrating lymphocytes (TILs), particularly CD4^+^ and CD8^+^ T cells and their immunomodulatory cytokines, are critical components of adaptive immunity within the TME (26, 27). The density of CD4^+^/CD8^+^ T cell infiltration in the tumor stroma is strongly associated with NSCLC patient prognosis, with higher TIL levels correlating with improved overall survival (OS) and disease-free survival (DFS) (28). CD8^+^ T cells, in particular, play a pivotal role in suppressing lung cancer progression, though their antitumor activity can be inhibited by Foxp3^+^ regulatory T cells (Tregs) (29). The transcriptional profiles of these cells offer insights for immunotherapy optimization and biomarker identification for checkpoint blockade therapies (30). Inhibitory receptors such as PD-1, TIM-3, CTLA-4, and LAG-3 on CD8^+^ T cells are linked to disease progression, as their overexpression impairs T cell activation and effector function (31). Ren et al. (32) demonstrated that PD-L1 expression in lung squamous cell carcinoma correlates with CD8^+^ TIL infiltration and MET gene upregulation, highlighting potential therapeutic targets.

CD4^+^ T cell subsets, including Tregs and Th17 cells, also significantly influence cancer pathogenesis (33–35). Tregs modulate cytokine and chemokine production, affecting immune cell recruitment and antitumor responses, thereby contributing to lung cancer progression (36). NSCLC patients exhibit higher CD4^+^ T cell frequencies in tumors compared to normal tissues, with CD4^+^Foxp3^+^ Tregs constituting a substantial TIL proportion (37). Cho et al. (11) identified Treg heterogeneity based on TNFRSF9 expression, linking activated Tregs to poor prognosis in lung adenocarcinoma. Th17 cells, characterized by IL-17 secretion, promote inflammation and lung cancer development, with IL-17 pathway alterations and genetic/epigenetic modifications increasing cancer susceptibility (12). Further research is needed to clarify the Treg-Th17 balance in lung cancer pathogenesis and its prognostic implications (38).

#### 2.2.2 Natural killer cells

**Figure 1:**
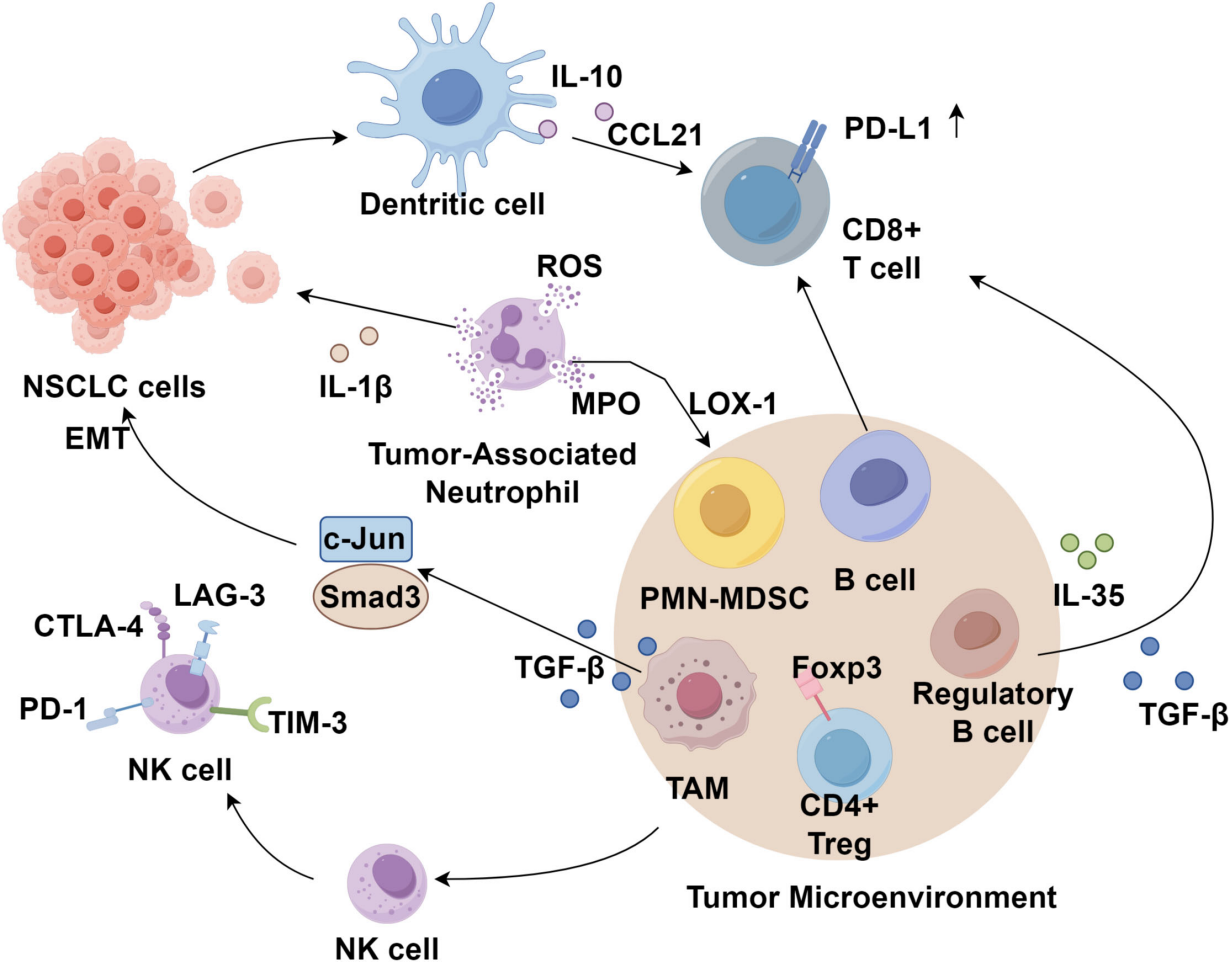
Immune cells in the NSCLC tumor immune microenvironment.

NK cells are critical early effectors in anti-tumor immunity and constitute the first line of defense against malignancies (39). The activation and cytotoxic function of NK cells are tightly regulated by a balance between inhibitory and activating receptors expressed on their surface (40). Studies have indicated that the presence of NK cells does not significantly impact the clinical outcomes of NSCLC patients, possibly due to the TME’s capacity to remodel intratumoral NK cells by reducing the expression of activating receptors while upregulating inhibitory receptors such as CTLA-4 (41). Additionally, NK cells express immune checkpoint molecules, including PD-1, LAG-3, and TIM-3 (42). Emerging evidence suggests that the lung TME may induce NK cell suppression, with CD8^+^ T cell infiltration positively correlating with CTLA-4 expression in NK cells, implying the presence of an inhibitory NK cell population even in immunologically active tumors (43). Despite significant advancements in cancer immunotherapy, a substantial proportion of patients fail to derive clinical benefit, partly due to the absence of a tumor-specific NK cell response. Therefore, strategies aimed at reactivating NK cells in combination with other therapeutic modalities may offer promising prospects for improving lung cancer treatment and prognosis (**Figure 1**).

### 2.3 Immunosuppressive and regulatory immune cells

#### 2.3.1 Myeloid-derived suppressor cells

MDSCs are a heterogeneous population of bone marrow-derived cells with potent immunosuppressive properties, widely present in the peripheral blood and tumor tissues of cancer patients (44–46). Their abundance and proportion correlate with tumor size and malignancy. MDSCs suppress both antigen-specific antitumor immunity mediated by T cells and non-specific antitumor immunity mediated by NK cells and macrophages through the expression of high levels of arginase, nitric oxide synthase, and reactive oxygen species (47). MDSCs are classified into polymorphonuclear MDSCs (PMN-MDSCs) and monocytic MDSCs (M-MDSCs). PMN-MDSCs exhibit morphological and phenotypic similarities to neutrophils, whereas M-MDSCs resemble immature monocytes. In both murine and human tumors, PMN-MDSCs predominate and closely resemble neutrophils in phenotype (48). Clinical analyses indicate that the expression of C-C chemokine receptor 5 (CCR5) is significantly elevated in peripheral blood M-MDSCs of NSCLC patients compared to healthy individuals. Additionally, the proportion of PMN-MDSCs and CCR5^+^ M-MDSCs in circulation negatively correlates with recurrence-free survival but is not associated with their proportion in tumor tissues (49). Tian et al. (50) demonstrated that endoplasmic reticulum stress in neutrophils induces significant upregulation of lectin-type oxidized LDL receptor-1 (LOX-1), facilitating their transformation into PMN-MDSCs. Current evidence suggests that the abundance of LOX-1^+^ PMN-MDSCs in NSCLC patients correlates with tumor size. However, further studies are warranted to evaluate their potential as prognostic biomarkers and therapeutic targets in NSCLC.

#### 2.3.2 Tumor-associated macrophages

Macrophages infiltrating the tumor tissue undergo differentiation and maturation into tumor-associated macrophages (TAMs) under the influence of immunosuppressive cytokines derived from both the tumor and its microenvironment (51). TAMs play a crucial role in promoting tumor angiogenesis and lymphangiogenesis (52). TAMs exhibit distinct phenotypic polarization, primarily classified into M1 and M2. IL-4 can induce M2 polarization of macrophages within the TME, contributing to extracellular matrix (ECM) degradation and TME remodeling (53). Lung cancer cells also promote M2 polarization of TAMs, and the secretion of TGF-β by TAMs activates the c-Jun and Smad3 pathways in lung cancer cells, leading to increased expression of SOX9, thereby facilitating epithelial-mesenchymal transition (EMT), tumor proliferation, migration, and invasion (54). A study by Alessandra et al. (55) demonstrated that the density of M2-polarized TAMs in the stroma of lung squamous cell carcinoma is significantly higher than that in lung adenocarcinoma. Furthermore, the density of M2 TAMs in the stroma of NSCLC correlates with lymph node metastasis, pathological staging, reduced disease-free survival (DFS), and OS (56, 57). These findings suggest that the stromal density of M2 TAMs serves as a potential indicator of tumor malignancy and clinical prognosis.

#### 2.3.3 Tumor-associated neutrophils

At the cellular level, neutrophils constitute the most abundant immune cell population in NSCLC, accounting for approximately 20% of total immune cells (58). Studies have indicated that early-stage lung cancer can induce the formation of a distinct subset of tumor-associated neutrophils (TANs) with APC characteristics, capable of cross-presenting antigens and enhancing anti-tumor T cell responses (59). TANs also contribute to tumor proliferation, invasion, and metastasis through the synthesis and secretion of specific proteases. McLoed et al. (60) demonstrated that neutrophils facilitate lung cancer progression by expressing IL-1β, thereby mediating resistance to nuclear factor-kappa B (NF-κB) inhibitors. This mechanism represents a key factor in the limited therapeutic efficacy of NF-κB inhibitors in lung cancer. Contrary to these pro-tumorigenic effects, neutrophils also exert anti-tumor and anti-metastatic functions (61). The primary mechanism by which TANs inhibit tumor cell proliferation involves antibody-dependent cellular cytotoxicity (ADCC), wherein antibodies recognize tumor cells via Fc receptors on TANs, leading to the release of cytotoxic mediators and subsequent tumor cell lysis. Moreover, TANs directly produce cytotoxic mediators such as reactive oxygen species (ROS) and myeloperoxidase (MPO), thereby inhibiting tumor cell proliferation (62). Studies have confirmed that interferon-beta (IFN-β) suppresses the expression of angiogenic factors such as vascular endothelial growth factor (VEGF) and matrix metalloproteinase-9 (MMP-9) in tumor-infiltrating neutrophils, leading to accelerated tumor vascularization and growth in IFN-β-deficient models (63). However, as most research on the role of neutrophils in lung cancer is based on animal models, limited data are available regarding the phenotype and function of TANs in lung cancer patients, necessitating further investigation.

### 2.4 Functional plasticity of tumor-infiltrating immune cells

The tumor microenvironment (TME) is characterized by the remarkable plasticity of infiltrating immune cells, which can adopt either anti-tumorigenic or pro-tumorigenic phenotypes depending on local signals, cytokine milieu, and tumor-derived factors (64). Effector CD8^+^ T cells mediate cytotoxicity against tumor cells but may become functionally exhausted, marked by sustained expression of inhibitory receptors, such as PD-1, TIM-3, LAG-3 and impaired cytokine production (65, 66). Similarly, macrophages exhibit phenotypic polarization along an M1–M2 spectrum: M1 macrophages promote antigen presentation and tumor clearance, whereas M2 macrophages support immunosuppression, angiogenesis, and metastasis (67, 68). B cells, dendritic cells, neutrophils, and NK cells also show phenotypic duality in NSCLC (69–71). This functional versatility enables tumors to co-opt immune responses and contributes to immunotherapy resistance (**Table 1**).

**Table 1:** Functional plasticity of key immune cells in the NSCLC tumor microenvironment.

Immune Cell Type	Anti-tumorigenic Phenotype	Pro-tumorigenic Phenotype
CD8^+^ T Cells	Effector T cells (high IFN-γ, granzyme B, cytotoxic activity)	Exhausted T cells (PD-1^+^, LAG-3^+^, TIM-3^+^, reduced cytotoxicity)
CD4^+^ T Cells	Th1 cells (support CD8^+^ T cell activation)	Tregs (Foxp3^+^, suppress immune response via IL-10, TGF-β)
B Cells	Antigen-presenting B cells, antibody-producing plasma cells	Regulatory B cells (IL-35, TGF-β secretion, Treg promotion)
Macrophages	M1 macrophages (IL-12^+^, TNF-α^+^, anti-tumor)	M2 macrophages (IL-10^+^, TGF-β^+^, promote EMT, angiogenesis)
Dendritic Cells	Mature DCs (high CD80/CD86, IL-12 secretion, antigen presentation)	Tolerogenic DCs (IL-10^+^, low co-stimulatory molecules)
Neutrophils	N1 TANs (antigen-presenting, promote CD8^+^ responses, ADCC)	N2 TANs (secrete VEGF, MMPs, promote metastasis)
NK Cells	Activated NK (NKG2D^+^, perforin/granzyme production)	Dysfunctional NK (PD-1^+^, CTLA-4^+^, impaired cytotoxicity)

### 2.5 Immune–stromal crosstalk and extracellular matrix remodeling

In NSCLC, immune cell–mediated modulation of the extracellular matrix (ECM) and stromal compartment plays a pivotal role in tumor invasion, angiogenesis, and immune evasion (72, 73). Tumor-associated macrophages (TAMs), particularly of the M2 phenotype, secrete matrix metalloproteinases (MMPs) such as MMP-9 and MMP-2, which degrade collagen and other ECM components, thereby facilitating tumor cell migration and metastasis (74–76). Additionally, TAM-derived transforming growth factor-beta (TGF-β) stimulates cancer-associated fibroblasts (CAFs), promoting desmoplastic reactions and the deposition of fibrotic ECM, which impairs immune cell infiltration and fosters an immunosuppressive niche (77–80). Neutrophils contribute similarly by releasing neutrophil elastase, enzymes that remodel ECM and release growth factors stored in the matrix (81, 82). CD8^+^ T cells and Th17 cells can also indirectly influence stromal remodeling through cytokines such as IFN-γ and IL-10, respectively, which modulate fibroblast activation and angiogenesis (66, 83). These immune–stromal interactions generate a dynamic feedback loop that alters the biophysical and biochemical properties of the TME, thereby enhancing immune resistance and diminishing immunotherapy efficacy (84). Understanding and targeting the immune-mediated reprogramming of the stroma represents a promising avenue for improving immunotherapeutic outcomes in NSCLC.

## 3 Immunotherapy for lung cancer

### 3.1 Monotherapy with immune checkpoint inhibitors

Immune checkpoint inhibitors have emerged as a cornerstone of first-line treatment for advanced NSCLC with high PD-L1 expression and no actionable driver mutations. Pembrolizumab, atezolizumab, and cemiplimab as monotherapy options have been approved for clinical evidence (85). The landmark KEYNOTE-024 trial established pembrolizumab as a superior alternative to platinum-based chemotherapy, demonstrating significant improvements in objective response rate (ORR), progression-free survival (PFS), and overall survival (OS) in patients with PD-L1 TPS ≥50% (86, 87). Long-term follow-up confirmed sustained survival benefits, reinforcing its role as a first-line standard of care. The subsequent KEYNOTE-042 trial expanded eligibility to patients with PD-L1 TPS ≥1%, though the greatest survival advantage remained confined to those with PD-L1 TPS ≥50% (88, 89). Further validation came from IMpower110 and EMPOWER-Lung 1, which demonstrated that atezolizumab and cemiplimab, respectively, significantly improved survival outcomes compared to chemotherapy in PD-L1-high populations (90–93). These findings collectively underscore the efficacy of ICI monotherapy in patients with high PD-L1 expression. In patients with high PD-L1 expression, atezolizumab and cemiplimab monotherapy significantly improved survival outcomes compared to standard chemotherapy. These findings underscore the transformative impact of immune monotherapy in PD-L1-high expressors; however, its efficacy in patients with low or absent PD-L1 expression remains limited. Therefore, combination immunotherapy strategies hold significant potential in expanding the patient population that may benefit from immune-based treatments.

### 3.2 Combination immunotherapy

Immunotherapy combined with chemotherapy is the guideline-recommended first-line treatment for advanced NSCLC without driver mutations, regardless of PD-L1 expression. The KEYNOTE-189 trial (94, 95) demonstrated that pembrolizumab plus chemotherapy significantly improved PFS and overall survival (OS) in non-squamous NSCLC compared to chemotherapy alone. The KEYNOTE-407 trial (95, 96) extended these benefits to squamous NSCLC, establishing pembrolizumab-chemotherapy as a preferred regimen for both subtypes. Similarly, the EMPOWER-Lung 3 study (97, 98) showed cemiplimab plus chemotherapy improved OS, leading to FDA approval. The IMpower130 trial (99) also supported atezolizumab-chemotherapy for non-squamous NSCLC, with OS benefits. In China, the National Medical Products Administration (NMPA) approved camrelizumab, sintilimab, toripalimab, and sugemalimab combined with chemotherapy based on trials such as CameL (100, 101), CameL-sq (102), ORIENT-11 (103, 104), ORIENT-12 (105), RATIONALE-304 (106), RATIONALE-307 (107, 108), and GEMSTONE-302 (109). Toripalimab-chemotherapy was approved for non-squamous NSCLC based on the CHOICE-01 study (110), while penpulimab and serplulimab were approved for squamous NSCLC based on AK105-302 (111) and ASTRUM-004 (112) trials, respectively. Combination immunotherapy with anti-angiogenic agents has also shown promise. The IMpower150 trial (113, 114) demonstrated that atezolizumab plus bevacizumab, carboplatin, and paclitaxel (ABCP) improved PFS and OS compared to bevacizumab-chemotherapy (BCP), leading to FDA approval for metastatic non-squamous NSCLC. Dual immunotherapy regimens combining PD-1/PD-L1 inhibitors with CTLA-4 inhibitors have also demonstrated efficacy. These regimens have been FDA-approved for advanced NSCLC based on their clinical trial outcomes.

## 4 Conclusion

A deeper understanding of the NSCLC immune microenvironment has yielded critical insights into how distinct immune cell populations can both foster and restrain tumor growth. T cells, in particular CD8^+^ T cells, remain central to tumor elimination, but their function is frequently hampered by immunosuppressive cells and inhibitory checkpoint pathways. B cells, dendritic cells, and natural killer cells similarly exhibit dual, context-dependent roles, underscoring the complexity of anti-tumor immunity. Myeloid-derived suppressor cells, tumor-associated macrophages, and neutrophils further highlight the TME’s capacity to promote immune evasion, metastasis, and therapy resistance. Recent clinical advances in immunotherapy have transformed the therapeutic paradigm for advanced NSCLC. Immune checkpoint inhibitors-alone or in combination with chemotherapy, anti-angiogenic agents, or other immunotherapeutic approaches-have significantly prolonged survival in select patient subgroups. Nevertheless, challenges remain, as many patients exhibit primary or acquired resistance, and the limited durability of responses underscores the need for more effective, personalized strategies.

We propose that a novel therapeutic paradigm should integrate immune contexture profiling, functional plasticity mapping of immune cells, and adaptive treatment strategies to convert immunosuppressive milieus into immunoreactive states. This review highlights that reprogramming key immune subsets—such as exhausted CD8^+^ T cells, M2-like TAMs, and suppressive MDSCs—may represent a transformative approach in NSCLC treatment. Subsequent investigations must prioritize the discovery of robust biomarkers for prediction, elucidating pathways underlying immunosuppressive phenomena and tumor immune evasion, as well as optimizing combined therapeutic protocols that enhance immune checkpoint inhibition efficacy. Through a comprehensive strategy incorporating mechanistic understanding of immunocyte behavior, malignant transformation processes, and innovative treatment frameworks, the oncology community can advance toward attaining sustained clinical responses and enhanced patient outcomes in late-stage non-small cell lung carcinoma.

## References

[1] Park AC Puchi C Austell P Lorch JH Alexiev BA Gharzai LA. Primary cutaneous neuroendocrine tumour of the scalp with metastasis. BMJ Case Rep. (2025) 18. doi: 10.1136/bcr-2024-263869, PMID: 40579201

[2] Alorfi F Bomanji J Bertoletti L Fraioli F. PET/MRI in non-small cell lung cancer (NSCLC). Semin Nucl Med. (2025) 55:234–9. doi: 10.1053/j.semnuclmed.2025.02.009, PMID: 40050130

[3] Bray F Ferlay J Soerjomataram I Siegel RL Torre LA Jemal A. Global cancer statistics 2018: GLOBOCAN estimates of incidence and mortality worldwide for 36 cancers in 185 countries. CA Cancer J Clin. (2018) 68:394–424. doi: 10.3322/caac.21492, PMID: 30207593

[4] Meyer ML Fitzgerald BG Paz-Ares L Cappuzzo F Jänne PA Peters S. New promises and challenges in the treatment of advanced non-small-cell lung cancer. Lancet. (2024) 404:803–22. doi: 10.1016/S0140-6736(24)01029-8, PMID: 39121882

[5] Desai A Peters S. Immunotherapy-based combinations in metastatic NSCLC. Cancer Treat Rev. (2023) 116:102545. doi: 10.1016/j.ctrv.2023.102545, PMID: 37030062

[6] Diao M Wang Y Wu S He S Liao Y. Estrogen, estrogen receptor and the tumor microenvironment of NSCLC. Int J Cancer. (2025) 156:1501–8. doi: 10.1002/ijc.35309, PMID: 39754298

[7] Zhao Y Chen X Huang Y Zhang Z Wang K Zou D. Transcriptomic insights into hub genes, immune infiltration, and candidate drugs in erosive esophagitis. J Inflammation Res. (2024) 17:7745–60. doi: 10.2147/JIR.S479032, PMID: 39494202 PMC11529285

[8] Liu R Wang K Guo X Wang Q Zhang X Peng K. A causal relationship between distinct immune features and acute or chronic pancreatitis: results from a mendelian randomization analysis. Pancreatology. (2024) 24:1219–28. doi: 10.1016/j.pan.2024.10.006, PMID: 39419750

[9] Hu Y Wang K Chen Y Jin Y Guo Q Tang H. Causal relationship between immune cell phenotypes and risk of biliary tract cancer: evidence from Mendelian randomization analysis. Front Immunol. (2024) 15:1430551. doi: 10.3389/fimmu.2024.1430551, PMID: 39050844 PMC11266158

[10] Chen C Guo Q Liu Y Hou Q Liao M Guo Y. Single-cell and spatial transcriptomics reveal POSTN(+) cancer-associated fibroblasts correlated with immune suppression and tumour progression in non-small cell lung cancer. Clin Transl Med. (2023) 13:e1515. doi: 10.1002/ctm2.1515, PMID: 38115703 PMC10731139

[11] Wang K Chen X Lin P Wu J Huang Q Chen ZN. CD147-K148me2-driven tumor cell-macrophage crosstalk provokes NSCLC immunosuppression via the CCL5/CCR5 axis. Adv Sci (Weinh). (2024) 11:e2400611. doi: 10.1002/advs.202400611, PMID: 38873823 PMC11304266

[12] Lin M Chen D Shao Z Liu Q Hao Z Xin Z. Inflammatory dendritic cells restrain CD11b(+)CD4(+) CTLs via CD200R in human NSCLC. Cell Rep. (2024) 43:113767. doi: 10.1016/j.celrep.2024.113767, PMID: 38354085

[13] Yim J Koh J Kim S Song SG Ahn HK Kim YA. Effects of B7-H3 expression on tumour-infiltrating immune cells and clinicopathological characteristics in non-small-cell lung cancer. Eur J Cancer. (2020) 133:74–85. doi: 10.1016/j.ejca.2020.03.033, PMID: 32447027

[14] Tian L Wang W Yu B Zhang G. Efficacy of dendritic cell-cytokine induced killer cells combined with concurrent chemoradiotherapy on locally advanced non-small cell lung cancer. J buon. (2020) 25:2364–70., PMID: 33277857

[15] Hou Y Zang D Li X Li F. Effect of cytokine-induced killer cells combined with dendritic cells on the survival rate and expression of 14-3-3ζ and p-Bad proteins in Lewis lung cancer cell lines. Oncol Lett. (2018) 16:1815–20. doi: 10.3892/ol.2018.8834, PMID: 30008870 PMC6036427

[16] Lee JM Lee MH Garon E Goldman JW Salehi-Rad R Baratelli FE. Phase I trial of intratumoral injection of CCL21 gene-modified dendritic cells in lung cancer elicits tumor-specific immune responses and CD8(+) T-cell infiltration. Clin Cancer Res. (2017) 23:4556–68. doi: 10.1158/1078-0432.CCR-16-2821, PMID: 28468947 PMC5599263

[17] Lu Y Xu W Gu Y Chang X Wei G Rong Z. Non-small cell lung cancer cells modulate the development of human CD1c(+) conventional dendritic cell subsets mediated by CD103 and CD205. Front Immunol. (2019) 10:2829. doi: 10.3389/fimmu.2019.02829, PMID: 31921114 PMC6914740

[18] Kasikova L Rakova J Hensler M Lanickova T Tomankova J Pasulka J. Tertiary lymphoid structures and B cells determine clinically relevant T cell phenotypes in ovarian cancer. Nat Commun. (2024) 15:2528. doi: 10.1038/s41467-024-46873-w, PMID: 38514660 PMC10957872

[19] Hou L Zhang S Yu W Yang X Shen M Hao X. Single-cell transcriptomics reveals tumor-infiltrating B cell function after neoadjuvant pembrolizumab and chemotherapy in non-small cell lung cancer. J Leukoc Biol. (2024) 116:555–64. doi: 10.1093/jleuko/qiad138, PMID: 37931147

[20] Germain C Devi-Marulkar P Knockaert S Biton J Kaplon H Letaïef L. Tertiary lymphoid structure-B cells narrow regulatory T cells impact in lung cancer patients. Front Immunol. (2021) 12:626776. doi: 10.3389/fimmu.2021.626776, PMID: 33763071 PMC7983944

[21] Berthe J Poudel P Segerer FJ Jennings EC Ng F Surace M. Exploring the impact of tertiary lymphoid structures maturity in NSCLC: insights from TLS scoring. Front Immunol. (2024) 15:1422206. doi: 10.3389/fimmu.2024.1422206, PMID: 39376565 PMC11457083

[22] Li S Mirlekar B Johnson BM Brickey WJ Wrobel JA Yang N. STING-induced regulatory B cells compromise NK function in cancer immunity. Nature. (2022) 610:373–80. doi: 10.1038/s41586-022-05254-3, PMID: 36198789 PMC9875944

[23] Lo Tartaro D Aramini B Masciale V Paschalidis N Lofaro FD Neroni A. Metabolically activated and highly polyfunctional intratumoral VISTA(+) regulatory B cells are associated with tumor recurrence in early-stage NSCLC. Mol Cancer. (2025) 24:16. doi: 10.1186/s12943-024-02209-2, PMID: 39810191 PMC11730485

[24] Chien CH Chiang BL. Regulatory T cells induced by B cells: a novel subpopulation of regulatory T cells. J BioMed Sci. (2017) 24:86. doi: 10.1186/s12929-017-0391-3, PMID: 29151021 PMC5694621

[25] Harris RJ Willsmore Z Laddach R Crescioli S Chauhan J Cheung A. Enriched circulating and tumor-resident TGF-β(+) regulatory B cells in patients with melanoma promote FOXP3(+) Tregs. Oncoimmunology. (2022) 11:2104426. doi: 10.1080/2162402X.2022.2104426, PMID: 35909944 PMC9336482

[26] Wang Y Liu M Zhang L Liu X Ji H Wang Y. Cancer CD39 drives metabolic adaption and mal-differentiation of CD4(+) T cells in patients with non-small-cell lung cancer. Cell Death Dis. (2023) 14:804. doi: 10.1038/s41419-023-06336-4, PMID: 38062068 PMC10703826

[27] Xie H Xi X Lei T Liu H Xia Z. CD8(+) T cell exhaustion in the tumor microenvironment of breast cancer. Front Immunol. (2024) 15:1507283. doi: 10.3389/fimmu.2024.1507283, PMID: 39717767 PMC11663851

[28] Bai Z Cheng X Ma T Li G Wang X Wang Z. CD8+ T cells infiltrating into tumors were controlled by immune status of pulmonary lymph nodes and correlated with non-small cell lung cancer (NSCLC) patients’ prognosis treated with chemoimmunotherapy. Lung Cancer. (2024) 197:107991. doi: 10.1016/j.lungcan.2024.107991, PMID: 39454350

[29] Ganesan AP Johansson M Ruffell B Yagui-Beltrán A Lau J Jablons DM. Tumor-infiltrating regulatory T cells inhibit endogenous cytotoxic T cell responses to lung adenocarcinoma. J Immunol. (2013) 191:2009–17. doi: 10.4049/jimmunol.1301317, PMID: 23851682 PMC3774528

[30] Chow A Uddin FZ Liu M Dobrin A Nabet BY Mangarin L. The ectonucleotidase CD39 identifies tumor-reactive CD8(+) T cells predictive of immune checkpoint blockade efficacy in human lung cancer. Immunity. (2023) 56:93–106.e106. doi: 10.1016/j.immuni.2022.12.001, PMID: 36574773 PMC9887636

[31] Dan A Aricak O Rounis K Montero-Fernandez MA Guijarro R Ekman S. PD-1 expression in tumor infiltrating lymphocytes as a prognostic marker in early-stage non-small cell lung cancer. Front Oncol. (2024) 14:1414900. doi: 10.3389/fonc.2024.1414900, PMID: 39391244 PMC11464330

[32] Ren W Xu Z Chang Y Ju F Wu H Liang Z. Pharmaceutical targeting of OTUB2 sensitizes tumors to cytotoxic T cells via degradation of PD-L1. Nat Commun. (2024) 15:9. doi: 10.1038/s41467-023-44466-7, PMID: 38167274 PMC10761827

[33] Liu Y Qin D Fu J. T lymphocyte heterogeneity in NSCLC: implications for biomarker development and therapeutic innovation. Front Immunol. (2025) 16:1604310. doi: 10.3389/fimmu.2025.1604310, PMID: 40510347 PMC12159075

[34] Sutanto H Ningtyas MC Rachma B Pratiwi L Fetarayani D. Th17 cells in cancer: plasticity-driven immunopathology and therapeutic opportunity. Immunol Cell Biol. (2025). doi: 10.1111/imcb.70043, PMID: 40569161

[35] Duan MC Zhong XN Liu GN Wei JR. The Treg/Th17 paradigm in lung cancer. J Immunol Res. (2014) 2014:730380. doi: 10.1155/2014/730380, PMID: 24872958 PMC4020459

[36] Gelibter A Asquino A Strigari L Zizzari IG Tuosto L Scirocchi F. CD137(+) and regulatory T cells as independent prognostic factors of survival in advanced non-oncogene addicted NSCLC patients treated with immunotherapy as first-line. J Transl Med. (2024) 22:329. doi: 10.1186/s12967-024-05142-6, PMID: 38570798 PMC10993529

[37] Devi-Marulkar P Fastenackels S Karapentiantz P Goc J Germain C Kaplon H. Regulatory T cells infiltrate the tumor-induced tertiary lymphoïd structures and are associated with poor clinical outcome in NSCLC. Commun Biol. (2022) 5:1416. doi: 10.1038/s42003-022-04356-y, PMID: 36566320 PMC9789959

[38] Kaabachi W ben Amor A Kaabachi S Rafrafi A Tizaoui K Hamzaoui K. Interleukin-17A and -17F genes polymorphisms in lung cancer. Cytokine. (2014) 66:23–9. doi: 10.1016/j.cyto.2013.12.012, PMID: 24548421

[39] Delconte RB Owyong M Santosa EK Srpan K Sheppard S McGuire TJ. Fasting reshapes tissue-specific niches to improve NK cell-mediated anti-tumor immunity. Immunity. (2024) 57:1923–1938.e1927. doi: 10.1016/j.immuni.2024.05.021, PMID: 38878769 PMC11684419

[40] Patel SA Nilsson MB Yang Y Le X Tran HT Elamin YY. IL6 mediates suppression of T- and NK-cell function in EMT-associated TKI-resistant EGFR-mutant NSCLC. Clin Cancer Res. (2023) 29:1292–304. doi: 10.1158/1078-0432.CCR-22-3379, PMID: 36595561 PMC10290888

[41] Bates AM Brown RJ Pieper AA Zangl LM Arthur I Carlson PM. Combination of bempegaldesleukin and anti-CTLA-4 prevents metastatic dissemination after primary resection or radiotherapy in a preclinical model of non-small cell lung cancer. Front Oncol. (2021) 11:645352. doi: 10.3389/fonc.2021.645352, PMID: 33937052 PMC8083981

[42] Datar I Sanmamed MF Wang J Henick BS Choi J Badri T. Expression analysis and significance of PD-1, LAG-3, and TIM-3 in human non-small cell lung cancer using spatially resolved and multiparametric single-cell analysis. Clin Cancer Res. (2019) 25:4663–73. doi: 10.1158/1078-0432.CCR-18-4142, PMID: 31053602 PMC7444693

[43] Russick J Joubert PE Gillard-Bocquet M Torset C Meylan M Petitprez F. Natural killer cells in the human lung tumor microenvironment display immune inhibitory functions. J Immunother Cancer. (2020) 8. doi: 10.1136/jitc-2020-001054, PMID: 33067317 PMC7570244

[44] Liang M Sun Z Chen X Wang L Wang H Qin L. E3 ligase TRIM28 promotes anti-PD-1 resistance in non-small cell lung cancer by enhancing the recruitment of myeloid-derived suppressor cells. J Exp Clin Cancer Res. (2023) 42:275. doi: 10.1186/s13046-023-02862-3, PMID: 37865804 PMC10589970

[45] Jeong H Koh J Kim S Yim J Song SG Kim H. Cell-intrinsic PD-L1 signaling drives immunosuppression by myeloid-derived suppressor cells through IL-6/Jak/Stat3 in PD-L1-high lung cancer. J Immunother Cancer. (2025) 13. doi: 10.1136/jitc-2024-010612, PMID: 40050048 PMC11887297

[46] Deng Y Shi M Yi L Naveed Khan M Xia Z Li X. Eliminating a barrier: Aiming at VISTA, reversing MDSC-mediated T cell suppression in the tumor microenvironment. Heliyon. (2024) 10:e37060. doi: 10.1016/j.heliyon.2024.e37060, PMID: 39286218 PMC11402941

[47] Wang Y Jia A Bi Y Wang Y Yang Q Cao Y. Targeting myeloid-derived suppressor cells in cancer immunotherapy. Cancers (Basel). (2020) 12. doi: 10.3390/cancers12092626, PMID: 32942545 PMC7564060

[48] Kobayashi T Nagata M Hachiya T Wakita H Ikehata Y Takahashi K. Increased circulating polymorphonuclear myeloid-derived suppressor cells are associated with prognosis of metastatic castration-resistant prostate cancer. Front Immunol. (2024) 15:1372771. doi: 10.3389/fimmu.2024.1372771, PMID: 38887300 PMC11180772

[49] Pant A Hwa-Lin Bergsneider B Srivastava S Kim T Jain A Bom S. CCR2 and CCR5 co-inhibition modulates immunosuppressive myeloid milieu in glioma and synergizes with anti-PD-1 therapy. Oncoimmunology. (2024) 13:2338965. doi: 10.1080/2162402X.2024.2338965, PMID: 38590799 PMC11000615

[50] Tian X Wang T Zheng Q Tao Y Dai L Shen H. Circulating CD15(+) LOX-1(+) PMN-MDSCs are a potential biomarker for the early diagnosis of non-small-cell lung cancer. Int J Clin Pract. (2021) 75:e14317. doi: 10.1111/ijcp.14317, PMID: 33960078

[51] Liu Y Yu Y Hu C Jiang M Zhao C Li X. ZEB2 upregulation modulates the polarization of TAMs toward the immunosuppressive state in EGFR-TKI-resistant NSCLC. Cancer Drug Resist. (2025) 8:25. doi: 10.20517/cdr.2024.206, PMID: 40510028 PMC12159605

[52] Xiao B Li G Gulizeba H Liu H Sima X Zhou T. Choline metabolism reprogramming mediates an immunosuppressive microenvironment in non-small cell lung cancer (NSCLC) by promoting tumor-associated macrophage functional polarization and endothelial cell proliferation. J Transl Med. (2024) 22:442. doi: 10.1186/s12967-024-05242-3, PMID: 38730286 PMC11084143

[53] Gao HX Liu MH Fan M Zhou JJ Li AQ Chen MW. MiR-135a-5p/STAT6-mediated EMT regulates IL-4 secretion in non-small cell lung cancer to affect M2-like TAM polarization. Int Immunopharmacol. (2025) 155:114623. doi: 10.1016/j.intimp.2025.114623, PMID: 40199139

[54] Zhang S Che D Yang F Chi C Meng H Shen J. Tumor-associated macrophages promote tumor metastasis via the TGF-β/SOX9 axis in non-small cell lung cancer. Oncotarget. (2017) 8:99801–15. doi: 10.18632/oncotarget.21068, PMID: 29245941 PMC5725132

[55] Rigamonti A Viatore M Polidori R Rahal D Erreni M Fumagalli MR. Integrating AI-powered digital pathology and imaging mass cytometry identifies key classifiers of tumor cells, stroma, and immune cells in non-small cell lung cancer. Cancer Res. (2024) 84:1165–77. doi: 10.1158/0008-5472.CAN-23-1698, PMID: 38315789 PMC10982643

[56] Schmid S Csanadi A Kozhuharov N Tchudjin M Kayser C Rawluk J. CC-chemokine ligand 18 is an independent prognostic marker in lymph node-positive non-small cell lung cancer. Anticancer Res. (2018) 38:3913–8. doi: 10.21873/anticanres.12676, PMID: 29970512

[57] Sumitomo R Hirai T Fujita M Murakami H Otake Y Huang CL. M2 tumor-associated macrophages promote tumor progression in non-small-cell lung cancer. Exp Ther Med. (2019) 18:4490–8. doi: 10.3892/etm.2019.8068, PMID: 31777551 PMC6862535

[58] Mitchell KG Lee Y Deboever N Negrao MV Tran HT Parra E. Haymaker CL: Intratumoral neutrophil-to-lymphocyte ratio is mirrored by circulating neutrophil-to-lymphocyte ratio in non-small cell lung cancer. J Immunother Cancer. (2025) 13., PMID: 40555561 10.1136/jitc-2025-011458 PMC12198785

[59] Singhal S Bhojnagarwala PS O’Brien S Moon EK Garfall AL Rao AS. Origin and role of a subset of tumor-associated neutrophils with antigen-presenting cell features in early-stage human lung cancer. Cancer Cell. (2016) 30:120–35. doi: 10.1016/j.ccell.2016.06.001, PMID: 27374224 PMC4945447

[60] McLoed AG Sherrill TP Cheng DS Han W Saxon JA Gleaves LA. Neutrophil-derived IL-1β Impairs the efficacy of NF-κB inhibitors against lung cancer. Cell Rep. (2016) 16:120–32. doi: 10.1016/j.celrep.2016.05.085, PMID: 27320908 PMC4927403

[61] Hänggi K Li J Gangadharan A Liu X Celias DP Osunmakinde O. Interleukin-1α release during necrotic-like cell death generates myeloid-driven immunosuppression that restricts anti-tumor immunity. Cancer Cell. (2024) 42:2015–2031.e2011., PMID: 39577420 10.1016/j.ccell.2024.10.014 PMC11631672

[62] Chen CL Wang Y Huang CY Zhou ZQ Zhao JJ Zhang XF. IL-17 induces antitumor immunity by promoting beneficial neutrophil recruitment and activation in esophageal squamous cell carcinoma. Oncoimmunology. (2017) 7:e1373234., PMID: 29296528 10.1080/2162402X.2017.1373234 PMC5739574

[63] Andzinski L Kasnitz N Stahnke S Wu CF Gereke M von Köckritz-Blickwede M. Type I IFNs induce anti-tumor polarization of tumor associated neutrophils in mice and human. Int J Cancer. (2016) 138:1982–93. doi: 10.1002/ijc.29945, PMID: 26619320

[64] Tian H Wei R Xiao C Fan T Che Y Liu T. Tumor-derived KLK8 predicts inferior survival and promotes an immune-suppressive tumor microenvironment in lung squamous cell carcinoma. BMC Pulm Med. (2024) 24:53. doi: 10.1186/s12890-023-02770-4, PMID: 38273291 PMC10809653

[65] Guégan JP Peyraud F Dadone-Montaudie B Teyssonneau D Palmieri LJ Clot E. Analysis of PD1, LAG3, TIGIT, and TIM3 expression in human lung adenocarcinoma reveals a 25-gene signature predicting immunotherapy response. Cell Rep Med. (2024) 5:101831. doi: 10.1016/j.xcrm.2024.101831, PMID: 39591972 PMC11722093

[66] Miotto D Lo Cascio N Stendardo M Querzoli P Pedriali M De Rosa E. CD8+ T cells expressing IL-10 are associated with a favourable prognosis in lung cancer. Lung Cancer. (2010) 69:355–60. doi: 10.1016/j.lungcan.2009.12.012, PMID: 20089329

[67] Huang X Yu G Jiang X Shen F Wang D Wu S. ITGB4/GNB5 axis promotes M2 macrophage reprogramming in NSCLC metastasis. Int Immunopharmacol. (2025) 144:113564. doi: 10.1016/j.intimp.2024.113564, PMID: 39577216

[68] Ren W Hou J Yang C Wang H Wu S Wu Y. Extracellular vesicles secreted by hypoxia pre-challenged mesenchymal stem cells promote non-small cell lung cancer cell growth and mobility as well as macrophage M2 polarization via miR-21-5p delivery. J Exp Clin Cancer Res. (2019) 38:62. doi: 10.1186/s13046-019-1027-0, PMID: 30736829 PMC6367822

[69] Wu Z Zhou J Xiao Y Ming J Zhou J Dong F. CD20(+)CD22(+)ADAM28(+) B cells in tertiary lymphoid structures promote immunotherapy response. Front Immunol. (2022) 13:865596. doi: 10.3389/fimmu.2022.865596, PMID: 35634306 PMC9130862

[70] de Oliveira JB Silva SB Fernandes IL Batah SS Herrera AJR Cetlin A. Dendritic cell-based immunotherapy in non-small cell lung cancer: a comprehensive critical review. Front Immunol. (2024) 15:1376704. doi: 10.3389/fimmu.2024.1376704, PMID: 39308861 PMC11412867

[71] Arasanz H Bocanegra AI Morilla I Fernández-Irigoyen J Martínez-Aguillo M Teijeira L. Circulating low density neutrophils are associated with resistance to first line anti-PD1/PDL1 immunotherapy in non-small cell lung cancer. Cancers (Basel). (2022) 14., PMID: 36010840 10.3390/cancers14163846 PMC9406164

[72] Wu J Zhang Q Yang Z Xu Y Liu X Wang X. CD248-expressing cancer-associated fibroblasts induce non-small cell lung cancer metastasis via Hippo pathway-mediated extracellular matrix stiffness. J Cell Mol Med. (2024) 28:e70025. doi: 10.1111/jcmm.70025, PMID: 39164826 PMC11335579

[73] Zhao J Liu M Zhu C Li Z Liu Z Abulizi D. Cancer-associated fibroblasts and metabolic reprogramming predict pathologic response to neoadjuvant PD-1 blockade in resected non-small cell lung cancer. Cell Oncol (Dordr). (2025). doi: 10.1007/s13402-025-01074-5, PMID: 40358847 PMC12238122

[74] Zhai K Jiang N Wen JF Zhang X Liu T Long KJ. Overexpression of TWF1 promotes lung adenocarcinoma progression and is associated with poor prognosis in cancer patients through the MMP1 signaling pathway. J Thorac Dis. (2023) 15:2644–58. doi: 10.21037/jtd-23-395, PMID: 37324107 PMC10267903

[75] Chen Y Ouyang D Wang Y Pan Q Zhao J Chen H. EBV promotes TCR-T-cell therapy resistance by inducing CD163+M2 macrophage polarization and MMP9 secretion. J Immunother Cancer. (2024) 12. doi: 10.1136/jitc-2023-008375, PMID: 38886114 PMC11184188

[76] Korotkaja K Lapina D Rudevica Z Zajakina A. Macrophage transcriptomic alterations driven by alphavirus-based cancer immunotherapy vectors. J Immunol Res. (2025) 2025:6573891. doi: 10.1155/jimr/6573891, PMID: 40547518 PMC12181664

[77] Tang PC Chan MK Chung JY Chan AS Zhang D Li C. Hematopoietic transcription factor RUNX1 is essential for promoting macrophage-myofibroblast transition in non-small-cell lung carcinoma. Adv Sci (Weinh). (2024) 11:e2302203. doi: 10.1002/advs.202302203, PMID: 37967345 PMC10767400

[78] Lin SC Liao YC Chen PM Yang YY Wang YH Tung SL. Periostin promotes ovarian cancer metastasis by enhancing M2 macrophages and cancer-associated fibroblasts via integrin-mediated NF-κB and TGF-β2 signaling. J BioMed Sci. (2022) 29:109. doi: 10.1186/s12929-022-00888-x, PMID: 36550569 PMC9784270

[79] Perez-Penco M Lara de la Torre L Lecoq I Martinenaite E Andersen MH. TGFβ-specific T cells induced by a TGFβ-derived immune modulatory vaccine both directly and indirectly modulate the phenotype of tumor-associated macrophages and fibroblasts. J Immunother Cancer. (2024) 12. doi: 10.1136/jitc-2023-008405, PMID: 38417917 PMC10900378

[80] Li D Xia L Huang P Wang Z Guo Q Huang C. Cancer-associated fibroblast-secreted IGFBP7 promotes gastric cancer by enhancing tumor associated macrophage infiltration via FGF2/FGFR1/PI3K/AKT axis. Cell Death Discov. (2023) 9:17. doi: 10.1038/s41420-023-01336-x, PMID: 36681667 PMC9867714

[81] Yagi T Kagawa S Nogi S Taniguchi A Yoshimoto M Suemori K. Cancer-associated fibroblasts promote pro-tumor functions of neutrophils in pancreatic cancer via IL-8: potential suppression by pirfenidone. Cancer Immunol Immunother. (2025) 74:96. doi: 10.1007/s00262-025-03946-z, PMID: 39904796 PMC11794937

[82] Giese MA Hind LE Huttenlocher A. Neutrophil plasticity in the tumor microenvironment. Blood. (2019) 133:2159–67. doi: 10.1182/blood-2018-11-844548, PMID: 30898857 PMC6524564

[83] Karachaliou N Gonzalez-Cao M Crespo G Drozdowskyj A Aldeguer E Gimenez-Capitan A. Interferon gamma, an important marker of response to immune checkpoint blockade in non-small cell lung cancer and melanoma patients. Ther Adv Med Oncol. (2018) 10:1758834017749748. doi: 10.1177/1758834017749748, PMID: 29383037 PMC5784541

[84] Liu Y Liang J Zhang Y Guo Q. Drug resistance and tumor immune microenvironment: An overview of current understandings (Review). Int J Oncol. (2024) 65. doi: 10.3892/ijo.2024.5684, PMID: 39219258 PMC11387120

[85] Ettinger DS Wood DE Aisner DL Akerley W Bauman JR Bharat A. NCCN guidelines^®^ Insights: non-small cell lung cancer, version 2.2023. J Natl Compr Canc Netw. (2023) 21:340–50., PMID: 37015337 10.6004/jnccn.2023.0020

[86] Reck M Rodríguez-Abreu D Robinson AG Hui R Csőszi T Fülöp A. Pembrolizumab versus chemotherapy for PD-L1-positive non-small-cell lung cancer. N Engl J Med. (2016) 375:1823–33. doi: 10.1056/NEJMoa1606774, PMID: 27718847

[87] Reck M Rodríguez-Abreu D Robinson AG Hui R Csőszi T Fülöp A. Five-year outcomes with pembrolizumab versus chemotherapy for metastatic non-small-cell lung cancer with PD-L1 tumor proportion score ≥ 50. J Clin Oncol. (2021) 39:2339–49. doi: 10.1200/JCO.21.00174, PMID: 33872070 PMC8280089

[88] Mok TSK Wu YL Kudaba I Kowalski DM Cho BC Turna HZ. Pembrolizumab versus chemotherapy for previously untreated, PD-L1-expressing, locally advanced or metastatic non-small-cell lung cancer (KEYNOTE-042): a randomised, open-label, controlled, phase 3 trial. Lancet. (2019) 393:1819–30., PMID: 30955977 10.1016/S0140-6736(18)32409-7

[89] de Castro G Jr. Kudaba I Wu YL Lopes G Kowalski DM Turna HZ. Five-year outcomes with pembrolizumab versus chemotherapy as first-line therapy in patients with non-small-cell lung cancer and programmed death ligand-1 tumor proportion score ≥ 1% in the KEYNOTE-042 study. J Clin Oncol. (2023) 41:1986–91. 10.1200/JCO.21.02885 PMC10082298 36306479

[90] Herbst RS Giaccone G de Marinis F Reinmuth N Vergnenegre A Barrios CH. Atezolizumab for first-line treatment of PD-L1-selected patients with NSCLC. N Engl J Med. (2020) 383:1328–39., PMID: 32997907 10.1056/NEJMoa1917346

[91] Jassem J de Marinis F Giaccone G Vergnenegre A Barrios CH Morise M. Updated overall survival analysis from IMpower110: atezolizumab versus platinum-based chemotherapy in treatment-naive programmed death-ligand 1-selected NSCLC. J Thorac Oncol. (2021) 16:1872–82., PMID: 34265434 10.1016/j.jtho.2021.06.019

[92] Sezer A Kilickap S Gümüş M Bondarenko I Özgüroğlu M Gogishvili M. Cemiplimab monotherapy for first-line treatment of advanced non-small-cell lung cancer with PD-L1 of at least 50%: a multicentre, open-label, global, phase 3, randomised, controlled trial. Lancet. (2021) 397:592–604. 33581821 10.1016/S0140-6736(21)00228-2

[93] Özgüroğlu M Kilickap S Sezer A Gümüş M Bondarenko I Gogishvili M. First-line cemiplimab monotherapy and continued cemiplimab beyond progression plus chemotherapy for advanced non-small-cell lung cancer with PD-L1 50% or more (EMPOWER-Lung 1): 35-month follow-up from a mutlicentre, open-label, randomised, phase 3 trial. Lancet Oncol. (2023) 24:989–1001. 37591293 10.1016/S1470-2045(23)00329-7

[94] Gandhi L Rodríguez-Abreu D Gadgeel S Esteban E Felip E De Angelis F. Pembrolizumab plus chemotherapy in metastatic non-small-cell lung cancer. N Engl J Med. (2018) 378:2078–92. 10.1056/NEJMoa1801005 29658856

[95] Garassino MC Gadgeel S Speranza G Felip E Esteban E Dómine M. Pembrolizumab plus pemetrexed and platinum in nonsquamous non-small-cell lung cancer: 5-year outcomes from the phase 3 KEYNOTE-189 study. J Clin Oncol. (2023) 41:1992–8. doi: 10.1200/JCO.22.01989, PMID: 36809080 PMC10082311

[96] Paz-Ares L Luft A Vicente D Tafreshi A Gümüş M Mazières J. Pembrolizumab plus chemotherapy for squamous non-small-cell lung cancer. N Engl J Med. (2018) 379:2040–51. doi: 10.1056/NEJMoa1810865, PMID: 30280635

[97] Gogishvili M Melkadze T Makharadze T Giorgadze D Dvorkin M Penkov K. Cemiplimab plus chemotherapy versus chemotherapy alone in non-small cell lung cancer: a randomized, controlled, double-blind phase 3 trial. Nat Med. (2022) 28:2374–80. doi: 10.1038/s41591-022-01977-y, PMID: 36008722 PMC9671806

[98] Makharadze T Gogishvili M Melkadze T Baramidze A Giorgadze D Penkov K. Cemiplimab plus chemotherapy versus chemotherapy alone in advanced NSCLC: 2-year follow-up from the phase 3 EMPOWER-lung 3 part 2 trial. J Thorac Oncol. (2023) 18:755–68. doi: 10.1016/j.jtho.2023.03.008, PMID: 37001859

[99] West H McCleod M Hussein M Morabito A Rittmeyer A Conter HJ. Atezolizumab in combination with carboplatin plus nab-paclitaxel chemotherapy compared with chemotherapy alone as first-line treatment for metastatic non-squamous non-small-cell lung cancer (IMpower130): a multicentre, randomised, open-label, phase 3 trial. Lancet Oncol. (2019) 20:924–37. doi: 10.1016/S1470-2045(19)30167-6, PMID: 31122901

[100] Zhou C Chen G Huang Y Zhou J Lin L Feng J. Camrelizumab plus carboplatin and pemetrexed versus chemotherapy alone in chemotherapy-naive patients with advanced non-squamous non-small-cell lung cancer (CameL): a randomised, open-label, multicentre, phase 3 trial. Lancet Respir Med. (2021) 9:305–14. doi: 10.1016/S2213-2600(20)30365-9, PMID: 33347829

[101] Zhou C Chen G Huang Y Zhou J Lin L Feng J. Camrelizumab plus carboplatin and pemetrexed as first-line treatment for advanced nonsquamous NSCLC: extended follow-up of cameL phase 3 trial. J Thorac Oncol. (2023) 18:628–39. doi: 10.1016/j.jtho.2022.12.017, PMID: 36646210

[102] Ren S Chen J Xu X Jiang T Cheng Y Chen G. Camrelizumab plus carboplatin and paclitaxel as first-line treatment for advanced squamous NSCLC (CameL-sq): A phase 3 trial. J Thorac Oncol. (2022) 17:544–57. doi: 10.1016/j.jtho.2021.11.018, PMID: 34923163

[103] Yang Y Wang Z Fang J Yu Q Han B Cang S. Efficacy and Safety of Sintilimab Plus Pemetrexed and Platinum as First-Line Treatment for Locally Advanced or Metastatic Nonsquamous NSCLC: a Randomized, Double-Blind, Phase 3 Study (Oncology pRogram by InnovENT anti-PD-1-11). J Thorac Oncol. (2020) 15:1636–46. doi: 10.1016/j.jtho.2020.07.014, PMID: 32781263

[104] Zhang L Wang Z Fang J Yu Q Han B Cang S. Final overall survival data of sintilimab plus pemetrexed and platinum as First-Line treatment for locally advanced or metastatic nonsquamous NSCLC in the Phase 3 ORIENT-11 study. Lung Cancer. (2022) 171:56–60. doi: 10.1016/j.lungcan.2022.07.013, PMID: 35917647

[105] Zhou C Wu L Fan Y Wang Z Liu L Chen G. Sintilimab plus platinum and gemcitabine as first-line treatment for advanced or metastatic squamous NSCLC: results from a randomized, double-blind, phase 3 trial (ORIENT-12). J Thorac Oncol. (2021) 16:1501–11. doi: 10.1016/j.jtho.2021.04.011, PMID: 34048947

[106] Lu S Wang J Yu Y Yu X Hu Y Ai X. Tislelizumab plus chemotherapy as first-line treatment for locally advanced or metastatic nonsquamous NSCLC (RATIONALE 304): A randomized phase 3 trial. J Thorac Oncol. (2021) 16:1512–22. doi: 10.1016/j.jtho.2021.05.005, PMID: 34033975

[107] Wang J Lu S Yu X Hu Y Sun Y Wang Z. Tislelizumab plus chemotherapy vs chemotherapy alone as first-line treatment for advanced squamous non-small-cell lung cancer: A phase 3 randomized clinical trial. JAMA Oncol. (2021) 7:709–17. doi: 10.1001/jamaoncol.2021.0366, PMID: 33792623 PMC8017481

[108] Wang J Lu S Yu X Hu Y Zhao J Sun M. Tislelizumab plus chemotherapy versus chemotherapy alone as first-line treatment for advanced squamous non-small-cell lung cancer: final analysis of the randomized, phase III RATIONALE-307 trial. ESMO Open. (2024) 9:103727. doi: 10.1016/j.esmoop.2024.103727, PMID: 39461775 PMC11549530

[109] Zhou C Wang Z Sun M Cao L Ma Z Wu R. Sugemalimab versus placebo, in combination with platinum-based chemotherapy, as first-line treatment of metastatic non-small-cell lung cancer (GEMSTONE-302): 4-year outcomes from a double-blind, randomised, phase 3 trial. Lancet Oncol. (2025)., PMID: 40523368 10.1016/S1470-2045(25)00198-6

[110] Wang Z Wu L Li B Cheng Y Li X Wang X. Toripalimab plus chemotherapy for patients with treatment-naive advanced non-small-cell lung cancer: A multicenter randomized phase III trial (CHOICE-01). J Clin Oncol. (2023) 41:651–63. doi: 10.1200/JCO.22.00727, PMID: 36206498 PMC9870236

[111] Zhong H Sun S Chen J Wang Z Zhao Y Zhang G. First-line penpulimab combined with paclitaxel and carboplatin for metastatic squamous non-small-cell lung cancer in China (AK105-302): a multicentre, randomised, double-blind, placebo-controlled phase 3 clinical trial. Lancet Respir Med. (2024) 12:355–65. doi: 10.1016/S2213-2600(23)00431-9, PMID: 38309287

[112] Zhou C Hu Y Arkania E Kilickap S Ying K Xu F. A global phase 3 study of serplulimab plus chemotherapy as first-line treatment for advanced squamous non-small-cell lung cancer (ASTRUM-004). Cancer Cell. (2024) 42:198–208.e193. doi: 10.1016/j.ccell.2023.12.004, PMID: 38181795

[113] Socinski MA Jotte RM Cappuzzo F Orlandi F Stroyakovskiy D Nogami N. Atezolizumab for first-line treatment of metastatic nonsquamous NSCLC. N Engl J Med. (2018) 378:2288–301. doi: 10.1056/NEJMoa1716948, PMID: 29863955

[114] Socinski MA Nishio M Jotte RM Cappuzzo F Orlandi F Stroyakovskiy D. IMpower150 final overall survival analyses for atezolizumab plus bevacizumab and chemotherapy in first-line metastatic nonsquamous NSCLC. J Thorac Oncol. (2021) 16:1909–24. doi: 10.1016/j.jtho.2021.07.009, PMID: 34311108

